# Perspectives and pitfalls of microbiome research through home based fecal sampling: the Flemish Gut Flora Project experience

**DOI:** 10.1186/2049-3258-73-S1-P33

**Published:** 2015-09-17

**Authors:** Doris Vandeputte, Rianne Vanleeuwen, Gwen Falony, Marie Joossens, Jeroen Raes

**Affiliations:** 1 Katholieke Universiteit Leuven, Leuven, Belgium

## 

The set up of large-scale, longitudinally sampled cohorts for microbiome research is logistically challenging as sample quality highly depends on the applied storage conditions. Ideally, fecal material intended for microbiome monitoring needs to be frozen immediately after sampling in order to stop the growth of residing bacteria and to conserve baseline microbial abundances. Effective fecal sampling protocols should not only combine comprehensive collection and storage instructions, but also excel in simplicity and hygiene of sample handling to avoid creating a potential population selection. To meet these criteria, the Flemish Gut Flora Project (FGFP), a large-scale (n>5000) microbiome research project based in Flanders (Belgium), developed a home sampling, aliquotting, and freezing protocol that, in combination with a cold chain collection network, would generate high-quality samples for microbiome research, while at the same time reducing logistic and post-collection analysis expenses.

Here we evaluate the FGFP sampling procedure in order to share the lessons learned from this project and improve future fecal sampling methods. We investigate selection bias imposed by the recruitment and check drop out values of the different steps of the sampling procedure. Based on questionnaires and temperature-time data of a subset of the samples we evaluate the cold chain and identify pitfalls of this crucial step in fecal sample collection for microbiome research. Furthermore recommendations to improve fecal sampling user experience are deduced from the responses to a user experience questionnaire and options for better fecal sampling procedures that combine these insights with methods to reduce laboratory efforts are proposed.

